# Standard Non-Personalized Electric Field Modeling of Twenty Typical tDCS Electrode Configurations via the Computational Finite Element Method: Contributions and Limitations of Two Different Approaches

**DOI:** 10.3390/biology10121230

**Published:** 2021-11-25

**Authors:** Andrés Molero-Chamizo, Michael A. Nitsche, Carolina Gutiérrez Lérida, Ángeles Salas Sánchez, Raquel Martín Riquel, Rafael Tomás Andújar Barroso, José Ramón Alameda Bailén, Jesús Carlos García Palomeque, Guadalupe Nathzidy Rivera-Urbina

**Affiliations:** 1 Department of Clinical and Experimental Psychology, University of Huelva, 21007 Huelva, Spain; urielass65@hotmail.com (Á.S.S.); rafan@uhu.es (R.T.A.B.); alameda@uhu.es (J.R.A.B.); 2 Leibniz Research Centre for Working Environment and Human Factors, 44139 Dortmund, Germany; nitsche@ifado.de; 3 Department of Neurology, University Medical Hospital Bergmannsheil, 44789 Bochum, Germany; 4 Department of Psychology, University of Córdoba, 14071 Córdoba, Spain; carolinagutierrez.lerida@gmail.com (C.G.L.); raquelm1993@gmail.com (R.M.R.); 5 Histology Department, School of Medicine, Cadiz University and District Jerez Costa-N., Andalusian Health Service, 11003 Cádiz, Spain; jesusc.garci.sspa@juntadeandalucia.es; 6 Psychology Center, Faculty of Administrative Sciences, Autonomous University of Baja California, Ensenada 22890, Mexico; nathzidy.rivera@uabc.edu.mx

**Keywords:** current flow, current intensity, electric field, finite element method, tDCS

## Abstract

**Simple Summary:**

The magnitude, distribution, and characteristics of the electric field induced in the brain by application of transcranial electric stimulation depend on the electrode configuration used and the specific parameters of stimulation. An approach to calculate the generated electric field is the computational finite element method. This method enables simulation of the current spread and electric field strength according to the electrode configuration and stimulation parameters used. However, current approaches have several limitations, which constrain the application of tDCS in empirical research and clinical practice. In this study, we provide examples of standard model-based electric field simulations corresponding to motor, dorsolateral prefrontal, and posterior parietal cortex stimulation using twenty typical electrode configurations. Two different current flow-modeling tools were used to compare the results and determine possible differences between both procedures regarding the specificity, as well as the reliability of the estimates. The results were rather consistent between both simulations. Some modest differences of the simulated distribution and intensity of the electric fields between the results of the respective modeling approaches were identified, which might have functional significance and reveal the need to empirically validate these models. The non-availability of quantitative data about the precise electric field distribution beyond the cortical target is a common limitation of both methods, which limits the determination of the spatial specificity of the intervention. These findings help to define future directions of research that allow to exploit the full potential of standard simulation approaches in the field of non-invasive brain stimulation.

**Abstract:**

Transcranial direct current stimulation (tDCS) is a non-invasive brain stimulation procedure to modulate cortical excitability and related brain functions. tDCS can effectively alter multiple brain functions in healthy humans and is suggested as a therapeutic tool in several neurological and psychiatric diseases. However, variability of results is an important limitation of this method. This variability may be due to multiple factors, including age, head and brain anatomy (including skull, skin, CSF and meninges), cognitive reserve and baseline performance level, specific task demands, as well as comorbidities in clinical settings. Different electrode montages are a further source of variability between tDCS studies. A procedure to estimate the electric field generated by specific tDCS electrode configurations, which can be helpful to adapt stimulation protocols, is the computational finite element method. This approach is useful to provide a priori modeling of the current spread and electric field intensity that will be generated according to the implemented electrode montage. Here, we present standard, non-personalized model-based electric field simulations for motor, dorsolateral prefrontal, and posterior parietal cortex stimulation according to twenty typical tDCS electrode configurations using two different current flow modeling software packages. The resulting simulated maximum intensity of the electric field, focality, and current spread were similar, but not identical, between models. The advantages and limitations of both mathematical simulations of the electric field are presented and discussed systematically, including aspects that, at present, prevent more widespread application of respective simulation approaches in the field of non-invasive brain stimulation.

## 1. Introduction

In the last two decades, a growing number of publications have provided evidence for the potential of transcranial electric stimulation (tES) techniques to modulate cortical excitability and underlying brain functions [1,2]. Transcranial direct current stimulation (tDCS) is the most often used tES variant, and the neurophysiological and functional effects of this technique have been carefully investigated [3,4,5,6,7,8]. Conventional [9,10,11] and recently introduced multi-electrode tDCS approaches [12] have been shown to be safe and effective methods to alter cognitive functions in healthy humans. Likewise, tDCS has moderate beneficial effects in several clinical conditions, with minimal side effects, both in adults [13,14] and children [15]. Moreover, the application of weak currents by tDCS is an inexpensive and easy-to-use procedure for clinicians and expert neuroscientists, compared to other non-invasive brain stimulation approaches. All these characteristics make tDCS a valuable tool to explore and alter brain functions, as well as a promising therapeutic application in a wide range of neurological and psychiatric symptoms [16,17,18,19]. 

A drawback of tDCS effects is its outcome variability [20,21], a feature that it shares with other neuromodulatory brain stimulation techniques. Response variability of tDCS depends on multifold individual and life conditions [22]. Main factors that determine inter-individual variability of tDCS effects include anatomical differences of the head and brain between subjects [23]. Indeed, the resistance of the skull, skin, cerebrospinal fluid (CSF), and meninges determines electrical conductance, and thus the amount of current applied to the skin entering the brain [24,25,26,27,28,29,30]. Besides, the international 10/20 EEG system is a common procedure used for tDCS electrode placement, and notable anatomical differences may compromise cortical correspondence with this standard system. Different electrode configurations used to modulate specific brain targets are also a source of heterogeneity of tDCS results [31], and the intensity of the electric field induced in each case is a key factor determining these differences. Thus, gaining information about the electric field intensity in the brain, as well as current spread and electric field directionality, resulting from specific tDCS electrode montages and applied current intensities is useful to optimize doses and electrode configurations in tDCS studies. 

A method to calculate and simulate the intensity and directionality of the electric field, current density and spread induced in the brain by tDCS is the finite element model, which is based on magnetic resonance imaging (MRI) data to build anatomically realistic head models [32,33]. Several software packages have used this computational method to provide electric field modeling in standardized or individual brain models based on specific stimulation parameters (electrode size, shape, and location, polarity—anode/cathode—over the cortical target, and current intensity measured in mA) [34,35]. This approach is used to predict and analyze the electric field resulting from specific tDCS electrode configurations and, thus, to decide for the optimal protocol in terms of appropriate electric field strengths for the intended brain target, and sufficient specificity of stimulation [36,37,38]. In modeling tools based on standard head models, which use standard conductivity values, electrode sizes, electrode positions, and current intensity can be adjusted to achieve the targeted electric fields in the brain, which can be helpful to determine optimal stimulation parameters. The predicted electric field strengths could furthermore serve as a reference to minimize between-study variability [39,40,41,42,43]. Physical and physiological empirical validation of the predicted electric fields, including the transfer of these predictions to functional and physiological effects of tDCS [44,45], requires, however, further research. 

Considering this background, an area to be explored is the comparison of available standard modeling approaches to analyze the resulting electric field intensities and distributions when default values for respective simulations are taken, and thus to evaluate the predictive potential of these methods. Therefore, the objective of this work is to provide simulations of the electric field induced by twenty typical tDCS electrode configurations using two different current flow modeling tools based on standard head models. We explored simulations of tDCS targeted to three brain regions and compared the results gained by these tools. Respective model-based adjustments of stimulation protocols might be helpful to determine electrode configurations in future tDCS studies. This includes determination of the maximum intensity of the electric field generated in the region of interest (ROI) for each electrode configuration (as explored here for the primary motor cortex—M1, dorsolateral prefrontal cortex—DLPFC, and posterior parietal cortex—PPC), as well as the distribution of the electric field dependent on electrode position and size, and current intensity. These data provide a priori information about predicted electric field strengths and characteristics, and are a relevant example how to define tDCS electrode configurations for empirical studies. The consistency or inconsistency of the results obtained by both modeling approaches will furthermore help to make assumptions about the level of reliability of these approaches in general, and the need for further empirical validation to exploit their full potential in basic research and clinical domains in future. In short, the simulations provided in this study are practical examples delivering a foundation for discussing the state-of-the-art, to analyze important shortcomings, and develop future research directions.

## 2. Materials and Methods

The SimNIBS 3.1.2 free-software [46], which is based on the finite element method and T1-and T2-weighted magnetic resonance images, was used to calculate the intensity of the electric field and simulate the current flow derived from 20 typical tDCS electrode configurations [47,48]. This software is based on a standard head model and allows the calculation of directed electric field (norm E-field) intensities (V/m) [49]. The head model includes about 4 × 10^6^ tetrahedra and 7 × 10^5^ nodes and was automatically created using the headreco tool of SimNIBS 2.1 [46]. Default tissue isotropic conductivity values provided by SimNIBS for scalar brain anisotropy are 0.126 S/m for the white matter, 0.275 S/m for the grey matter, 1.654 S/m for the cerebrospinal fluid (CSF), 0.010 S/m for the bone (skull), 0.465 S/m for the scalp, 0.5 S/m for the eyes, and 1.0 S/m for the saline-soaked sponges [50]. Tissue segmentation is based on structural MRI data, conducted with the MeshFix tool [51], and the calculated direction of the electric field is normal to the local surface orientation [46,52]. 

The results from this simulation approach were compared with those of another electric field modeling tool based on the finite element method and a different head model: Computation of Electric field due to Transcranial current Stimulation (COMETS) v.2.0 [53]. Here, a four-layer human head model, including a cortical surface model composed of scalp, skull, cerebrospinal fluid, white and grey matter, is used as a default head model. An additional software, the ISO2MESH [54], is applied to provide tetrahedral meshes of the head model. The surface meshes of the four layers (scalp, outer skull boundary, inner skull boundary, and brain cortical surface) are extracted from structural MRI data [53]. Unlike SimNIBS, COMETS is a MATLAB-based toolbox, which automatically generates electrode pads, simulating conventional sponge-covered electrodes, whose sizes and angles can be adjusted. The electric field and current density values are scaled by the ratio of the target-injected current to the computed injected current passing through the bottom of the electrode. Electrical conductivity default values are 0.22 S/m for the grey matter, 1.79 S/m for the CSF, 0.014 S/m for the bone (skull), and 0.22 S/m for the scalp [53,55]. As with SimNIBS, tissue segmentation in COMETS is based on structural MRI data, and the computed direction of the electric field is normal to the surface orientation [53,56]. Thus, differences between both modeling software packages are based on the selected default MRI data, conductivity values and biological tissues, and the computational tool for algorithm calculation. Both approaches share the finite element method for mathematical calculation of the electric field, and the opportunity for freely setting the stimulation parameters (electrode size and position, and current intensity). For validation of the models, electric field predictions from computational modeling have been compared with empirical measurements in a few studies [57], including direct in vivo measures of the tDCS-induced electric field by intracranial recordings [44,58], and indirect neuroimaging approaches [59]. Intracranial measurements have shown the importance of some stimulation parameters, such as electrode placement [57], and the precision of tissue conductivity values of simulations [44], for the accuracy of electric field calculations. Validation of modeling results is still incomplete at present, and further improvements, such as controlling for the impact of intracranial electrodes required for obtaining electrical field data on the measurement results [60], should be taken into account for respective approximations in future studies. While validation of the physical electric field modeling faces respective limitations, indirect validations of the simulations with respect to physiological and behavioral effects also are incomplete. In general, these attempts show moderate associations between the simulated electric fields and several functional measures [45,61]. However, an exact quantification of these associations is not available at present and should be the topic of future studies. 

The twenty electrode configurations whose electric fields were modeled are shown in Table 1. They were taken from classical configurations aimed to target M1 [5,6], DLPFC [62], and PPC [63] as ROIs. The MRI coordinates (axis x, y, z) corresponding to the electrode position of each ROI in the COMETS simulation were: M1 (x = −62.069; y = 18.763; z = 112.696), DLPFC (x = −50.9573; y = 73.9931; z = 81.0353), and PPC (x = −46.52327; y = −42.38308; z = 123.114). The SimNIBS coordinates for the electrode position for each ROI were: M1 (x = −67.16; y = −16.60; z = 82.76), DLPFC (x = −49.33; y = 52.25; z = 73.03), and PPC (x = −54.66; y = −85.61; z = 64.84). These coordinates correspond to the specific MRIs used in each software to create the respective head model, in which the ROI can be localized via the international 10–20 EEG system for electrode positioning. Therefore, in this study we define the ROIs as cortical regions defined by standard electrode positions, in particular, the C3 electrode position for targeting the left M1 region in the frontal lobe, the F3 electrode position for the left DLPFC in the prefrontal area, and the P3 electrode position for the left PPC in the parietal lobe. 

We explored the results for different electrode sizes (5 cm × 4 cm = 20 cm^2^ vs. 7 cm × 5 cm = 35 cm^2^), return electrode positions (contralateral homologous region vs. right supraorbital region -rSOR), and current intensities (1 vs. 2 mA dose, for demonstrating the proportionality of the induced electric fields and the injected current). The combination of these parameters and cortical regions resulted in the following twenty electrode montages: (1–2) anodal left primary motor cortex (lM1; C3 electrode position according to the international 10–20 EEG system)-cathodal right supraorbital region (rSOR; Fp2 electrode position), at 1 and 2 mA intensity, respectively, and 20 cm^2^ electrode size; (3–4) anodal lM1-cathodal right M1 (C4 electrode position), at 1 and 2 mA, respectively, and 20 cm^2^ electrode size; (5–6) anodal lM1-cathodal rSOR, at 1 and 2 mA of intensity, respectively, and 35 cm^2^ electrode size; (7–8) anodal lM1- cathodal right M1, at 1 and 2 mA intensity, respectively, and 35 cm^2^ electrode size; (9–10) anodal left dorsolateral prefrontal cortex (lDLPFC; F3 electrode position)-cathodal rSOR, at 1 and 2 mA of intensity, respectively, and 20 cm^2^ electrode size; (11–12) anodal lDLPFC-cathodal right DLPFC (rDLPFC; F4 electrode position), at 1 and 2 mA of intensity, respectively, and 20 cm^2^ cm electrode size; (13–14) anodal lDLPFC-cathodal rSOR, at 1 and 2 mA of intensity, respectively, and 35 cm^2^ electrode size; (15–16) anodal lDLPFC-cathodal rDLPFC, at 1 and 2 mA of intensity, respectively, and 35 cm^2^ electrode size; (17–18) anodal left posterior parietal cortex (lPPC; P3 electrode position)-cathodal rSOR, at 1 and 2 mA of intensity, respectively, and 20 cm^2^ electrode size; (19–20) anodal lPPC-cathodal rSOR, at 1 and 2 mA of intensity, respectively, and 35 cm^2^ electrode size. 

We compared the intensities and distribution of the predicted electric fields between both modeling tools, according to electrode position, electrode size, and current intensity. The parameters of the electric fields evaluated for this purpose were defined as (i) the maximum intensity of the electric field within the ROI = the peak of intensity as measured by V/m; and (ii) the electric field distribution associated with each electrode configuration as a measure of specificity. Contrasting the results of different stimulation protocols allows to evaluate the characteristics of their resulting electric fields (peak of intensity and distribution), which provides information about the specificity of each protocol. Additionally, comparisons between different modeling approaches allow to analyze the consistency of the results, and therefore their reliability. The consistency or inconsistency of the simulation results between both models may serve as a guide to motivate further empirical validation of estimates based on standard models.

## 3. Results

**Figure 1:**
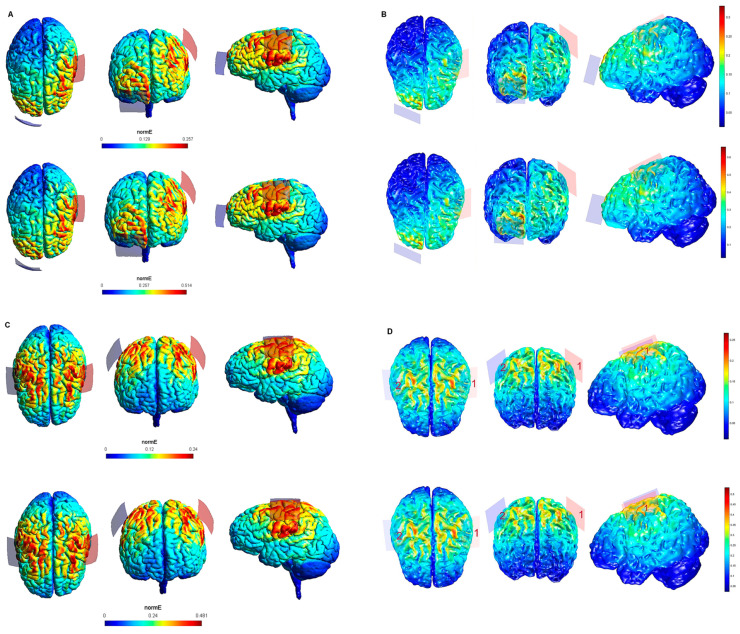
Electric field modeling corresponding to the tDCS electrode configurations 1–4 (M1 and 20 cm^2^ electrode size): 1–2, anodal left primary motor cortex (lM1)-cathodal right supraorbital region (rSOR), at 1 and 2 mA stimulation intensity, respectively, and 20 cm^2^ electrode size; 3–4, anodal lM1-cathodal right M1, at 1 and 2 mA stimulation intensity, respectively, and 20 cm^2^ electrode size. Dorsal, frontal, and lateral SimNIBS (**A**) and COMETS (**B**) outputs for configurations 1–2 at 1 (superior images) and 2 (inferior images) mA stimulation intensity are depicted. Dorsal, frontal, and lateral SimNIBS (**C**) and COMETS (**D**) outputs for configurations 3–4 at 1 (superior images) and 2 (inferior images) mA stimulation intensity are depicted. The normal electric field (normE) intensity (V/m) is represented by the color bar (online version). Brighter colors (corresponding to larger values in the color bar) indicate higher electric field intensity. Red and blue electrodes represent the anodal and cathodal electrode positions, respectively.

**Figure 2:**
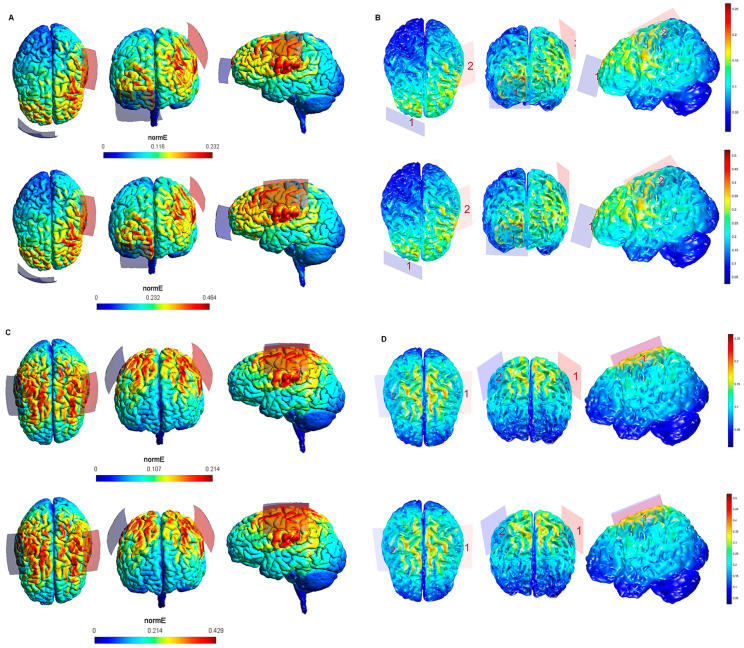
Electric field modeling corresponding to the tDCS electrode configurations 5–8 (M1 and 35 cm^2^ electrode size): 5–6, anodal lM1-cathodal rSOR, at 1 and 2 mA stimulation intensity, respectively, and 35 cm^2^ electrode size; 7–8, anodal lM1-cathodal right M1, at 1 and 2 mA stimulation intensity, respectively, and 35 cm^2^ electrode size. Dorsal, frontal and lateral SimNIBS (**A**) and COMETS (**B**) outputs for configurations 5–6 at 1 (superior images) and 2 (inferior images) mA stimulation intensity are depicted. Dorsal, frontal, and lateral SimNIBS (**C**) and COMETS (**D**) outputs for configurations 7–8 at 1 (superior images) and 2 (inferior images) mA stimulation intensity are depicted. For the color bar, electric field intensity, and electrodes information, refer to Figure 1.

**Figure 3:**
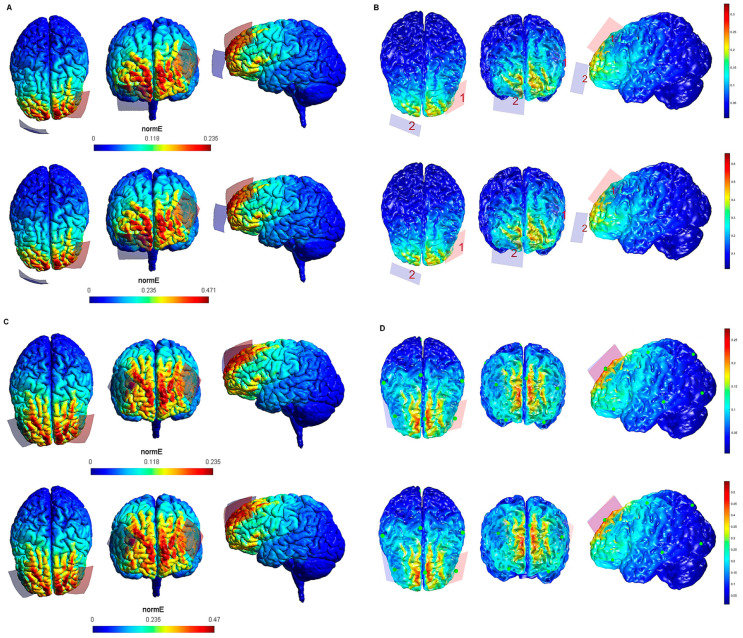
Electric field modeling corresponding to the tDCS electrode configurations 9–12 (DLPFC and 20 cm^2^ electrode size): 9–10, anodal left dorsolateral prefrontal cortex (lDLPFC)-cathodal rSOR, at 1 and 2 mA stimulation intensity, respectively, and 20 cm^2^ electrode size; 11–12, anodal lDLPFC-cathodal right DLPFC (rDLPFC), at 1 and 2 mA stimulation intensity, respectively, and 20 cm^2^ electrode size. Dorsal, frontal, and lateral SimNIBS (**A**) and COMETS (**B**) outputs for configurations 9–10 at 1 (superior images) and 2 (inferior images) mA stimulation intensity are shown. Dorsal, frontal and lateral SimNIBS (**C**) and COMETS (**D**) outputs for configurations 11–12 at 1 (superior images) and 2 (inferior images) mA stimulation intensity are depicted. For the color bar, electric field intensity, and electrodes information, refer to Figure 1.

**Figure 4:**
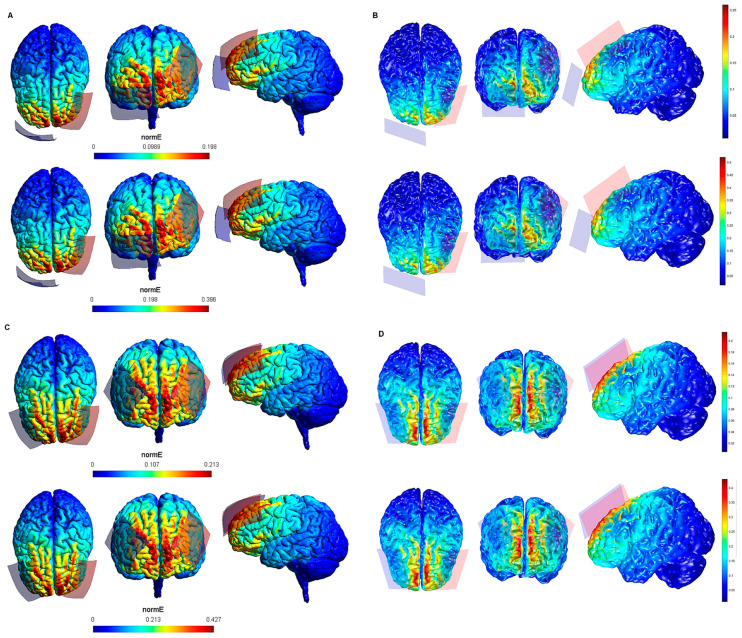
Electric field modeling corresponding to the tDCS electrode configurations 13–16 (DLPFC and 35 cm^2^ electrode size): 13–14, anodal lDLPFC-cathodal rSOR, at 1 and 2 mA current intensity, respectively, and 35 cm^2^ electrode size; 15–16 anodal lDLPFC-cathodal rDLPFC, at 1 and 2 mA stimulation intensity, respectively, and 35 cm^2^ electrode size. Dorsal, frontal and lateral SimNIBS (**A**) and COMETS (**B**) outputs for configurations 13–14 at 1 (superior images) and 2 (inferior images) mA stimulation intensity are depicted. Dorsal, frontal, and lateral SimNIBS (**C**) and COMETS (**D**) outputs for configurations 15–16 at 1 (superior images) and 2 (inferior images) mA stimulation intensity are shown. For the color bar, electric field intensity, and electrodes information, see Figure 1.

**Figure 5:**
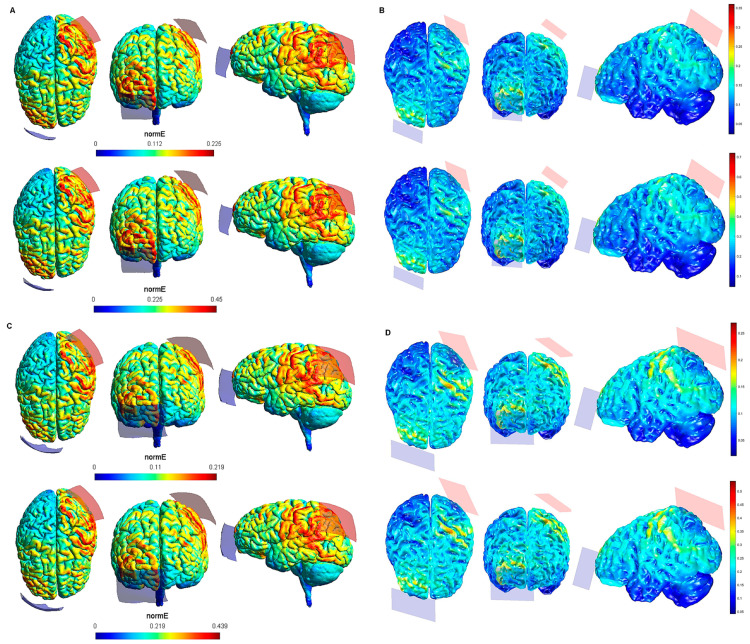
Electric field modeling corresponding to the tDCS electrode configurations 17–20 (PPC and 20/35 cm^2^ electrode size): 17–18, anodal left posterior parietal cortex (lPPC)-cathodal rSOR, at 1 and 2 mA stimulation intensity, respectively, and 20 cm^2^ electrode size; 19–20, anodal lPPC-cathodal rSOR, at 1 and 2 mA stimulation intensity, respectively, and 35 cm^2^ electrode size. Dorsal, frontal, and lateral SimNIBS (**A**) and COMETS (**B**) outputs for configurations 17–18 at 1 (superior images) and 2 (inferior images) mA stimulation intensity are shown. Dorsal, frontal, and lateral SimNIBS (**C**) and COMETS (**D**) outputs for configurations 19–20 at 1 (superior images) and 2 (inferior images) mA stimulation intensity are depicted. For the color bar, electric field intensity, and electrodes information, refer to Figure 1.

The maximum intensity values of the electric field generated in the brain for each tDCS electrode configuration based on both software packages are shown in Table 1. The maximum intensity of the electric field in the brain corresponded to the peak of intensity over the respective ROIs (i). Figure 1, Figure 2, Figure 3, Figure 4 and Figure 5 shows the SimNIBS and COMETS normal electric field modeling results corresponding to the electrode configurations 1–4, 5–8, 9–12, 13–16, and 17–20, respectively. 

The spatial distribution of the electric fields (ii) is shown qualitatively by the outputs of both modeling approaches, via whole brain cortical surface images. Variations of the color of the electric field indicate the range of electrical field strengths. Different from the peak electric field intensity, which is quantified by the models, electric field distributions can only be reported qualitatively, based on the outputs of the respective modeling software packages. However, based on the images, we provide estimated ranges of electric field intensity (by reporting lowest and highest estimated intensities for each area) in cortical regions impacted by the current beyond the ROI for each electrode configuration and modeling approach as a measure of electric field distribution (Table 2). In general, electric field intensities outside the ROIs were higher when using tDCS configurations with 2 mA and 20 cm^2^ electrode sizes, as compared to lower stimulation intensity, and larger electrode sizes. Considering that the electric field strength induced by a direct current that has been linked to moderate physiological effects on slices of animal brain tissue, i.e., effects on neuronal ion channels and excitability, is about 5 V m^−1^ (=0.2 V/m) [64,65,66,67], a description of the areas outside the respective ROIs clearly exposed to with such intensity might help to establish the potentially physiologically relevant electric field distribution beyond the cortical target. When the M1 was the target, the areas outside the ROI in which the lowest intensity of the electric field reached this threshold (>0.2 V/m, according to both SimNIBS and COMETS simulations, Table 2), were restricted to the premotor cortex (PMC) (configuration 2: lM1/rSOR-20 cm^2^-2 mA), ventromedial prefrontal cortex (vmPFC) (configuration 2: lM1/rSOR-20 cm^2^-2 mA), dorsomedial prefrontal cortex (dmPFC) (configuration 2: lM1/rSOR-20 cm^2^-2 mA), and DLPFC (configuration 2: lM1/rSOR-20 cm^2^-2 mA). With the same cortical target, electric field intensity also reached this threshold under the electrode configuration 6 (lM1/rSOR-35 cm^2^-2 mA), but only in the dmPFC, and under the configuration 4 (lM1/rM1-20 cm^2^-2 mA), in the anterior parietal cortex (APC). When stimulation was targeting the DLPFC (Table 2), electric field intensity reached this threshold in the vmPFC and dmPFC with the electrode configurations 12 (lDLPFC/rDLPF-20 cm^2^-2 mA) and 16 (lDLPFC/rDLPF-35 cm^2^-2 mA), and only in the vmPFC under the configurations 10 (lDLPFC/rSOR-20 cm^2^-2 mA), and 14 (lDLPFC/rSOR-35 cm^2^-2 mA). When targeting the DLPFC, electric field intensity within the orbitofrontal cortex (OFC) reached this threshold under the electrode configurations 14 (lDLPFC/rSOR-35 cm^2^-2 mA) and 16 (lDLPFC/rDLPF-35 cm^2^-2 mA). No electrode configuration targeting the PPC was associated to electric field intensities above this threshold outside the ROI (Table 2).

According to the qualitative distribution of the electric field observed in each brain image corresponding to each electrode configuration, the electric field reached its peak intensity over the M1 in both modeling approaches for the electrode configurations 1–2 (lM1-rSOR, stimulation intensity 1–2 mA, electrode size 20 cm^2^). The electric field was extended beyond the ROI (M1) in both cases, although the extension was slightly larger in the SimNIBS simulation (Figure 1A), compared to COMETS (Figure 1B). When the electrode montages involved both M1 (configurations 3–4: anode over the left, and cathode over the right motor cortex), both modeling approaches showed similarly distributed electric fields throughout the motor regions of both hemispheres (Figure 1C,D). The extension of the electric field beyond the target was slightly larger in the homologous configurations with larger electrode sizes (35 cm^2^ vs. 20 cm^2^) (electrode configurations 5–8; Figure 2), although the distribution pattern (involving frontal regions beyond M1 and anterior parietal regions) was comparable between these electrode configurations. In the electrode configurations 9–12, the electric field covered the ROI (lDLPFC, Figure 3) and was more focused to this target when the cathodal electrode was positioned over the rSOR (Fp2; configurations 9–10), in comparison with the rDLPFC (F4; configurations 11–12), as particularly shown by the COMETS simulation (Figure 3B). With larger electrode sizes (35 cm^2^) (electrode configurations 13–16; Figure 4), both simulation tools showed lower peak intensities, but a similar electric field distribution, as compared to the smaller electrodes. The respective maximum intensities were also localized in the DLPFC, and more focally restricted with the cathode positioned over the rSOR (configurations 13–14), as compared to bilateral stimulation, according to both simulations, but with a larger difference suggested by the COMETS results (Figure 4B). For the electrode configurations 17–20 (Figure 5), aimed at stimulating the PPC region, again electric field intensity peaked in the intended cortical target. The cortical boundaries showing maximum electric field intensity were more restricted to the cortical region corresponding to the PPC (P3) with smaller electrode sizes (20 cm^2^) (Figure 5B), compared to the larger ones (35 cm^2^) (Figure 5D), as revealed by both simulations, but more clearly by the COMETS outputs. This difference between the cortical boundaries with maximum electric fields due to different electrode sizes was less clear-cut in the SimNIBS simulations (Figure 5A,C). 

Although the electrical field intensities peaked in respective cortical targets in all electrode configurations (Table 1), electric fields were also observed beyond the respective ROIs in all cases. None of the explored electrode configurations resulted in fields completely restricted to M1, DLPFC, or PPC (Figure 1, Figure 2, Figure 3, Figure 4 and Figure 5). Overall, the current spread associated to each electrode configuration was comparable between modeling approaches, although the COMETS simulations generally suggested less extensive spreading. The most striking difference between SimNIBS and COMETS outputs regarding current spread was found for configurations 17–20 (Figure 5). The smallest current spread beyond the PPC was found for configurations 17–18 (lPPC-rSOR, at 1–2 mA, 20 cm^2^), according to the COMETS outputs which revealed a minimum current spread (Figure 5B). However, the SimNIBS outputs revealed a more extended current spread from the lPPC, reaching temporal and frontal cortical areas (Figure 5A). The current spread associated with configurations 19–20 (35 cm^2^ electrode sizes) was somewhat larger compared to the homologous configurations using smaller electrodes, with SimNIBS showing again a wider current spread (Figure 5C). 

Overall, these results indicate that higher maximum electric field intensities were obtained by the COMETS simulation. On the other hand, less focal electrical fields were observed in general in SimNIBS modeling, especially with respect to simulations involving the PPC.

## 4. Discussion

The finite element method has been proposed as an approach to estimate the electric fields induced by specific tES electrode configurations [68,69]. Electrical neuromodulation protocols in clinical and research settings might benefit from this method [33,39,44,70]. Comparing two well-established free access software packages based on this calculation method, we calculated the peak intensity and distribution of the electric field resulting from twenty typical tDCS electrode configurations. Different cortical targets, electrode sizes, and current intensities were explored. Both modeling tools provided grossly comparable and congruent electric field simulation results (Table 1), although differences with respect to the topography of the spatial distribution and the magnitude of the electric fields were evident (Table 2). 

The findings of this study show that the maximum intensity of the electric field does not vary more than 0.086 V/m between the two modeling methods in the configurations targeting the M1, 0.129 V/m in configurations targeting the DLPFC, and 0.25 V/m in configurations targeting the PPC. Thus, the results reveal larger between-model differences regarding the electric field peak when the parietal cortex is stimulated, and smaller between-model differences in the intensity of the electric field when the M1 is stimulated. On the other hand, the results of SimNIBS simulations reveal an overall larger current spread compared to COMETS simulations, especially under configurations targeting the parietal cortex. This heterogeneity makes empirical validation of standard model-based electric field simulations especially important. The validation methods of the estimates might reveal possible underestimations or overestimations of electric fields, which is of particular value given that the functional and physiological effects of neuromodulation seem to depend on the characteristics of the current delivered to the brain [38,39,62,71].

In all configurations aimed to induce neuromodulation of M1 (1–8), minimal differences between the two simulation tools were found regarding maximum electric field intensities (differences up to 0.086 V/m, with constantly higher values from COMETS). Bilateral stimulation, involving both hemispheres, resulted in more focally restricted effects over M1, particularly for COMETS modeling. The typical left M1 (anode electrode)-right SOR (cathode electrode) configuration resulted in increased current spread from M1 towards prefrontal cortex regions in all cases. However, according to both tools, electric field intensity reached the level of 2 V/m outside the ROI only when applying 2 mA of current intensity. The frontal areas reaching this threshold under this electrode configuration and current intensity were the PMC, vmPFC, dmPFC, and DLPFC. With the bilateral configuration targeting the M1 (lM1-rM1), the threshold of 2 V/m outside the ROI was only reached with 2 mA of current intensity as well, but only in the APC. Similar electric field strengths over the ROI were obtained using both tools for bilateral (lM1-rM1) or unilateral (lM1-rSOR) M1 stimulation, with slightly higher maximum intensities in the unilateral configurations. The results of these electric field calculations for the target M1 can be contrasted with physiological effects of respective stimulation protocols on motor cortex excitability obtained via transcranial magnetic stimulation (TMS)-elicited motor-evoked potentials (MEP) [5,42,72,73,74,75,76], and with motor cortex activity data recorded by functional magnetic resonance imaging (fMRI) [61,77,78,79,80]. For the left M1-right SOR electrode montage, respective tDCS-induced excitability alterations have been shown by multiple studies [5,6,75,80], whereas for bilateral M1 stimulation, the situation is less clear in spite of similar modeled electrical field strengths. Therefore, the strength and distribution of electric fields do not necessarily translate one-to-one into established physiological effects [61,81]. A critical factor to be considered in both electrode montages (lM1-rM1 vs. lM1-rSOR), which could explain these differences between modeling as well as empirical results, might be that the alignment of each electric field and neuronal orientation has an important impact on physiological effects of this intervention [45,82,83,84], and this is not taken into consideration by these modeling approaches. Moreover, increased electric field intensities do not necessarily result in stronger physiological effects of stimulation [28,72]. Accordingly, for quantitatively predicting physiological effects of tDCS, physical modeling might not be sufficient without the control of these critical factors. 

Similar to the M1 modeling results, for all configurations involving the DLPFC, minimal differences between the two simulation tools were found with respect to the maximum electric field intensity (differences not larger than 0.129 V/m, with higher values mainly, but not exclusively, obtained by COMETS) as well, but an opposite pattern of electrode position-dependent focality (as compared to M1) was observed for configurations targeting this prefrontal region (9–16). Bilateral DLPFC stimulation (anode over the left DLPFC and cathode over the right DLPFC) was associated with larger current spread over the prefrontal cortex, particularly over ventromedial and dorsomedial regions, for all current intensities and electrode sizes. The extension of the current spread was larger for the SimNIBS modeling results. In the bilateral configuration targeting the DLPFC, the threshold of 2 V/m was exceeded outside the ROI in the vmPFC and dmPFC only when applying a current strength of 2 mA, according to both simulation tools, and only in the vmPFC in the case of the unilateral electrode configuration and a current intensity of 2 mA. For both electrode configurations, unilateral and bilateral, this threshold was reached in the OFC only with 2 mA current intensity. The left DLPFC (anode)–right SOR (cathode) configuration showed higher focality in all electrode size/intensity combinations. As compared to available evidence regarding the M1, the physiological changes induced in the DLPFC by tDCS are less well explored [85], which makes it difficult to establish a reasonable correspondence between the results of the electric field modeling and physiological results associated with neuromodulation of this cortical region [86]. 

The most notable differences were found for electrode configurations aimed to stimulate the left PPC (17–20). On the one hand, the largest difference between both simulation tools was found for the left PPC-right SOR configuration (2 mA, 20 cm^2^), in which the maximum electric field intensity was 0.25 V/m higher in the COMETS modeling. On the other hand, the focality of the SimNIBS-simulated electric field was similar for all electrode configurations, but evident between-configuration differences in electric field focality were found in COMETS simulations. With SimNIBS, current spread involved parietal, temporal, and frontal regions of the left hemisphere, while it was much more limited when the electric field was calculated by COMETS, with moreover larger focality associated with smaller electrodes (20 cm^2^). Despite these differences between models, with respect to current spread characteristics, the electric field intensity calculated by both methods did not reach the threshold of 2 V/m outside the PPC with any of the electrode configurations targeting this area. The overall strength and distribution of these electric fields align with physiological results showing increases of PPC excitability and electrical activity under similar tDCS protocols, as measured by TMS-elicited MEP [64] and event-related potentials [87,88]. The correspondence of these simulated electric fields with behavioral results induced by anodal stimulation of the PPC [88,89] is, however, less clear. 

Overall, while both simulation approaches revealed generally comparable results, higher values of the maximum electric field intensity resulted from the COMETS simulation (with only two exceptions, corresponding to the left DLPFC-right DLPFC configuration, 35 cm^2^ electrode size, at intensities of 1 and 2 mA respectively), while the SimNIBS results show less focality and larger current spread, particularly when the left PPC was the cortical target. The latter was specifically evident for the electric field computed for the left PPC-right SOR electrode montage, in which anode–cathode electrode distance was the largest of all modeled configurations. These differences might be caused by the different default conductivity values of both modeling approaches, whose effects on the outcomes will increase with larger distances between electrodes, and by the fact that SimNIBS integrates more tissues in respective models, as compared to COMETS. Assumptions about the relative validity of both modeling approaches would require face-to-face experimental approaches involving measures of electrical fields, but respective data are not available at present. The peak of electrical filed intensity was doubled in all electrode configurations when applying 2 mA of stimulation, as compared to 1 mA, which is trivial according to the quasi-static assumption for the electric fields induced by stimulation. 

Standardized non-individualized modeling approaches, as shown here, provide quantitative estimations of the peak electric field intensity, and a qualitative overview about electric field distributions achieved with conventional electrode montages. These results are valuable for gaining a general overview about the electrical fields induced by specific tES protocols, but face also some limitations. For validation of the predictions made by the models, a few studies are available [44,57,58,59], which show gross alignment of the modeled with the empirically obtained electrical fields. However, at least gradual discrepancies between predicted and empirically obtained electrical fields are described, and empirically validated evidence for the correctness of the models with respect to electrical field intensities at the target and non-target sites is incomplete at present [44,60,90,91]. These discrepancies might be due to the limited number of tissues included in currently available models [48], which might impair the numerical accuracy of the estimations [29,48]. Similarly, differences between SimNIBS and COMETS results might be caused, at least partially, by differences between these tools with respect to the included tissues. Additionally, as a common limitation of both methods, specific characteristics of the tDCS electrodes (such as shape, connector position, conductivities of saline, or electrode gel used) have recently been suggested as another determinant of the simulated electric fields [50], and not all of these can be freely set up for electric field modeling. The SimNIBS tool provides the opportunity to conduct TMS-induced electric field calculations as well, including individualized head models [92,93]. As for tDCS-induced electric field simulations, this approach also faces some limitations, including specific coil characteristics that can be set for modeling. Currently, modeling methods provide a priori information about the characteristics of the respective electric fields, and these estimates may be helpful for implementation of research protocols in the neuromodulation field. Approaches based on standard MR images might be suited to define physically optimal electrode positions, and stimulation intensities at the group level, which might however not result in optimal approaches at the level of individual. Approaches based on individual MRI, which are not discussed in detail here, might allow individual adaptations of stimulation protocols to achieve inter-individually homogeneous electrical fields in future. Likewise, in clinical settings, the main advantage of modeling for future applications may be the use of these models for personalized adaptation of stimulation parameters to reach sufficient electrical field strength in patients, considering the reported relationship between tDCS effects and the current delivered in the brain [45,61], rather than to discern responders from non-responders [94]. This would however require quantitatively reliable empirical validation of the simulations and of the suitability of this approach, which should be the topic of future studies. Based on such validated simulations, individually adapted modeling could enhance the efficacy and consistency of neuromodulation effects over different individuals [39,93,94]. 

Another limitation of these models is that these are purely based on macroscopic anatomy. They cannot take into account the alignment of electric fields with neuronal orientation in a detailed way, which might however be decisive for physiological and also functional effects [45,82,83,84]. Regarding decisions on electrode positions based on electric field simulations, it is moreover important to notice that not only the location of the maximum electric field intensity is an important factor, but also the specificity of the electrical field for the targeted cortical area. In this regard, the information that can be extracted from the versions of the models we included in the present study is limited, because no precise quantitative results of the electric field distribution are delivered. Respective modeling approaches are however available. 

At the level of the individual, gradual misalignment between predicted and empirical electrical fields in case of non-personalized modeling, as conducted here, can be caused by anatomical differences between the head model and individual brains [21,41,61,71,86,95]. Non-personalized models have thus limited exactness for individual brains. However, they may be suited for gaining an initial overview of the physical effects of stimulation. 

It should furthermore be noted that these models generate data about electrical fields, but not directly about physiological, psychological, or behavioral effects. Pure physical models do not necessarily predict cognitive and behavioral effects of an intervention. For improving the correspondence between simulated electric fields and cognitive and behavioral effects of tDCS, brain physiology, including task-related physiological alterations during stimulation, has to be considered. This is particularly relevant since task- [96,97,98,99] and brain state- [100] specific behavioral alterations accomplished by tDCS have been reported [101,102,103]. Simulated electric fields are thus valuable for the design of tDCS studies; it is however important to take into consideration that these are not the only relevant parameters. Attempts to relate some characteristics of simulated electric fields with cognitive functions have however been described [39,104]. 

As proposals for future studies, the use of modeling for multielectrode stimulation approaches, which may induce more spatially restricted electrical fields [105], may be interesting to explore the focality of different electrode configurations to a larger degree, although this was beyond the scope of this contribution. Another factor that requires to be considered in modeling tools is the reliability of modeling in specific populations, such as children [24,106] or patients with neurodegeneration or brain damage [107,108]. Due to anatomical specifics, here standard models based on healthy adult brains are likely not appropriate. 

In summary, standard modeling, as shown here, can be a useful approach for initial screening of tDCS protocols. However, since the resulting electric field estimations are not based on all potentially relevant anatomical factors, quantitative information about the spatial dispersion of the electrical fields is not available in these models, and simulations do not include physiological models, this strategy faces relevant limitations for making detailed decisions about stimulation protocols. Some fundamental aspects should be stressed in this regard. First, the simulation of the electric fields in these models is not based on individual brain data [109], and both tools used different head models. Thus, the default electric resistance, current conductivity, tissue properties, and gross head anatomy, were similar, but not identical, between models, which might have caused the slight differences in the results obtained by these simulations. Most simulation software packages, however, including the freely available models we used, allow now individualized electric field modeling based on individual head models obtained through neuroimaging [95,110,111]. A priori this is advantageous because it could improve the quality of respective models and might help to explain interindividual differences and personalize tDCS protocols in future. Nevertheless, individual neuroimaging is a demanding resource not always available, and might not solve the between subject variability phenomenon completely, with respect to physiological, psychological, and behavioral effects, because there is no one-to-one translation between the physical models and physiological results. Improving the alignment between physical modeling and physiological effects would probably require a control of neuronal orientation and electric field directionality, anatomical structures and physiological processes involved, which neither are readily available resources for modeling at present. Comparisons between the results obtained by different modeling software packages have not been reported before, and our approximation of quantification of the electric fields in the non-target regions is an effort to deliver relevant information that usually is not stressed in modeling. This novel approach allows thus an extended, systematic analysis of the potentials and limitations of different models, and modeling in general. Besides, the findings of this study might contribute to establish future directions of use of standard, non-personalized modeling methods.

## 5. Conclusions

We provide examples about how current flow can be calculated using two different modeling tools for tDCS configurations varying with respect to electrode positions, current intensity, and electrode sizes. Based on this approach, we discussed advantages and limitations of those simulations in which individual MRI is not required, with respect to basic experimental research and clinical applications. We furthermore discuss future perspectives of electric field simulations of non-invasive brain stimulation.

The general outcomes of the simulation procedures were relatively similar between models, although higher values of the maximum intensity of the electric field were generally obtained by COMETS modeling. Less focality and extended current spread were observed when the electric field was simulated by SimNIBS, particularly with those configurations in which the anode electrode was positioned over the PPC region. Between-model differences were thus apparent for quantitative measures of electric field intensity and qualitative overviews of the electric field distribution. However, it can be concluded that, in general, both simulation approaches deliver roughly comparable results for a standard head model. Unresolved issues of these modeling approaches are the limited physical and physiological validation of simulations, and the transfer of results to behavioral and clinical levels. These shortcomings do currently prevent the application of modeling for routine clinical applications, which would be however a principally important field for personalization of therapeutic neuromodulation in neuropsychiatric disorders to reduce interindividual variability of results [16,112]. A one-to-one translation from simulated electric fields to brain physiology is not currently feasible, given neuronal direction-sensitive effects and non-linear effects of stimulation, among other uncontrolled factors, although some physiological effects can be predicted based on modeling that use individual physical measures [28]. To control these factors in simulation to a larger degree, validated models at the microscale and physiological levels would be necessary. Currently available modeling procedures supply, however, also in case of simple, non-personalized approaches, valuable information for the development of tES studies, by providing a general overview of electric field characteristics. 

## Figures and Tables

**Table 1:** Electric field intensity calculated by the finite element method (SimNIBS 3.1.2 and COMETS v.2.0) for twenty typical tDCS electrode configurations.

Electrode Configuration	Anode Location	Cathode Location	Electrode Size	Current Intensity (V/m)	Current Intensity at the Electrode- Skin Interface	Maximum Electric Field in the Brain (SimNIBS—COMETS)
1	lM1	rSOR	5 cm × 4 cm (20 cm^2^)	1 mA	0.05 mA/cm^2^	0.257—0.3 V/m
2	lM1	rSOR	5 cm × 4 cm (20 cm^2^)	2 mA	0.1 mA/cm^2^	0.514—0.6 V/m
3	lM1	rM1	5 cm × 4 cm (20 cm^2^)	1 mA	0.05 mA/cm^2^	0.24—0.25 V/m
4	lM1	rM1	5 cm × 4 cm (20 cm^2^)	2 mA	0.1 mA/cm^2^	0.481—0.5 V/m
5	lM1	rSOR	7 cm × 5 cm (35 cm^2^)	1 mA	0.02 mA/cm^2^	0.232—0.25 V/m
6	lM1	rSOR	7 cm × 5 cm (35 cm^2^)	2 mA	0.05 mA/cm^2^	0.464—0.5 V/m
7	lM1	rM1	7 cm × 5 cm (35 cm^2^)	1 mA	0.02 mA/cm^2^	0.214—0.25 V/m
8	lM1	rM1	7 cm × 5 cm (35 cm^2^)	2 mA	0.05 mA/cm^2^	0.429—0.5 V/m
9	lDLPFC lDLPFC	rSOR	5 cm × 4 cm (20 cm^2^)	1 mA	0.05 mA/cm^2^	0.235—0.3 V/m
10	lDLPFC	rSOR	5 cm × 4 cm (20 cm^2^)	2 mA	0.1 mA/cm^2^	0.471—0.6 V/m
11	lDLPFC	rDLPFC	5 cm × 4 cm (20 cm^2^)	1 mA	0.05 mA/cm^2^	0.235—0.25 V/m
12	lDLPFC	rDLPFC	5 cm × 4 cm (20 cm^2^)	2 mA	0.1 mA/cm^2^	0.47—0.5 V/m
13	lDLPFC	rSOR	7 cm × 5 cm (35 cm^2^)	1 mA	0.02 mA/cm^2^	0.198—0.25 V/m
14	lDLPFC	rSOR	7 cm × 5 cm (35 cm^2^)	2 mA	0.05 mA/cm^2^	0.396—0.5 V/m
15	lDLPFC	rDLPFC	7 cm × 5 cm (35 cm^2^)	1 mA	0.02 mA/cm^2^	0.213—0.2 V/m
16	lPPC	rDLPFC	7 cm × 5 cm (35 cm^2^)	2 mA	0.05 mA/cm^2^	0.427—0.4 V/m
17	lPPC	rSOR	5 cm × 4 cm (20 cm^2^)	1 mA	0.05 mA/cm^2^	0.225—0.35 V/m
18	lPPC	rSOR	5 cm × 4 cm (20 cm^2^)	2 mA	0.1 mA/cm^2^	0.45—0.7 V/m
19	lPPC	rSOR	7 cm × 5 cm (35 cm^2^)	1 mA	0.02 mA/cm^2^	0.219—0.25 V/m
20		rSOR	7 cm × 5 cm (35 cm^2^)	2 mA	0.05 mA/cm^2^	0.439—0.5 V/m

l/rDLPFC, left/right dorsolateral prefrontal cortex (F3/F4, according to the international 10–20 EEG electrode positioning system); l/rM1, left/right primary motor cortex (C3/C4); lPPC, left posterior parietal cortex (P3); rSOR, right supraorbital ridge (Fp2).

**Table 2:** Estimated electric field strengths in the cortical regions of the left hemisphere impacted by the current as a measure of the electric field distribution beyond the ROI for each of the 20 tested tDCS electrode configurations and modeling methods (SimNIBS and COMETS).

**Electrode Configuration**	**PMC (S)**	**PMC (C)**	**vmPFC (S)**	**vmPFC (C)**	**dmPFC (S)**	**dmPFC (C)**
1 (lM1/rSOR-20 cm^2^-1 mA)	>0.129 <0.257 V/m	>0.1 <0.2 V/m	>0.05 <0.1 V/m	>0.08 <0.14 V/m	>0.1 <0.17 V/m	>0.13 <0.18 V/m
2 (lM1/rSOR-20 cm^2^-2 mA)	>0.257 <0.35 V/m	>0.25 <0.35 V/m	>0.2 <0.3 V/m	>0.24 <0.34 V/m	>0.25 <0.36 V/m	>0.24 <0.35 V/m
3 (lM1/rM1-20 cm^2^-1 mA)	>0.12 <0.17 V/m	>0.12 <0.15 V/m	>0.04 <0.08 V/m	>0.05 <0.1 V/m	>0.06 <0.17 V/m	>0.08 <0.14 V/m
4 (lM1/rM1-20 cm^2^-2 mA)	>0.15 <0.35 V/m	>0.16 <0.34 V/m	>0.1 <0.2 V/m	>0.12 <0.2 V/m	>0.09 <0.2 V/m	>0.12 <0.19 V/m
5 (lM1/rSOR-35 cm^2^-1 mA)	>0.07 <0.19 V/m	>0.09 <0.17 V/m	>0.08 <0.16 V/m	>0.09 <0.16 V/m	>0.09 <0.16 V/m	>0.11 <0.17 V/m
6 (lM1/rSOR-35 cm^2^-2 mA)	>0.17 <0.36 V/m	>0.19 <0.33 V/m	>0.17 <0.29 V/m	>0.19 <0.31 V/m	>0.2 <0.32 V/m	>0.2 <0.31 V/m
7 (lM1/rM1-35 cm^2^-1 mA)	>0.07 <0.17 V/m	>0.07 <0.17 V/m	>0.06 <0.11 V/m	>0.05 <0.12 V/m	>0.07 <0.13 V/m	>0.07 <0.13 V/m
8 (lM1/rM1-35 cm^2^-2 mA)	>0.15 <0.36 V/m	>0.15 <0.34 V/m	>0.11 <0.22 V/m	>0.11 <0.23 V/m	>0.15 <0.25 V/m	>0.15 <0.26 V/m
9 (lDLPFC/rSOR-20 cm^2^-1 mA)	>0.06 <0.15 V/m	>0.07 <0.14 V/m	>0.12 <0.2 V/m	>0.15 <0.24 V/m	>0.1 <0.21 V/m	>0.14 <0.22 V/m
10 (lDLPFC/rSOR-20 cm^2^-2 mA)	>0.12 <0.29 V/m	>0.12 <0.3 V/m	>0.25 <0.4 V/m	>0.29 <0.49 V/m	>0.18 <0.41 V/m	>0.25 <0.43 V/m
11 (lDLPFC/rDLPF-20 cm^2^-1 mA)	>0.07 <0.17 V/m	>0.07 <0.17 V/m	>0.14 <0.22 V/m	>0.14 <0.22 V/m	>0.1 <0.21 V/m	>0.15 <0.24 V/m
12 (lDLPFC/rDLPF-20 cm^2^-2 mA)	>0.14 <0.35 V/m	>0.12 <0.33 V/m	>0.29 <0.41 V/m	>0.27 <0.41 V/m	>0.23 <0.39 V/m	>0.29 <0.48 V/m
13 (lDLPFC/rSOR-35 cm^2^-1 mA)	>0.07 <0.13 V/m	>0.05 <0.11 V/m	>0.13 <0.19 V/m	>0.15 <0.19 V/m	>0.07 <0.17 V/m	>0.09 <0.19 V/m
14 (lDLPFC/rSOR-35 cm^2^-2 mA)	>0.13 <0.27 V/m	>0.1 <0.22 V/m	>0.25 <0.35 V/m	>0.32 <0.39 V/m	>0.14 <0.36 V/m	>0.19 <0.37 V/m
15 (lDLPFC/rDLPF-35 cm^2^-1 mA)	>0.07 <0.19 V/m	>0.04 <0.15 V/m	>0.17 <0.2 V/m	>0.13 <0.19 V/m	>0.12 <0.18 V/m	>0.13 <0.19 V/m
16 (lDLPFC/rDLPF-35 cm^2^-2 mA)	>0.14 <0.33 V/m	>0.09 <0.32 V/m	>0.32 <0.39 V/m	>0.28 <0.39 V/m	>0.24 <0.39 V/m	>0.27 <0.38 V/m
17 (lPPC/rSOR-20 cm^2^-1 mA)	>0.07 <0.17 V/m	>0.08 <0.15 V/m	>0.05 <0.14 V/m	>0.1 <0.17 V/m	>0.07 <0.17 V/m	>0.1 <0.16 V/m
18 (lPPC/rSOR-20 cm^2^-2 mA)	>0.16 <0.34 V/m	>0.18 <0.3 V/m	>0.12 <0.26 V/m	>0.19 <0.35 V/m	>0.18 <0.29 V/m	>0.18 <0.34 V/m
19 (lPPC/rSOR-35 cm^2^-1 mA)	>0.08 <0.17 V/m	>0.08 <0.13 V/m	>0.06 <0.15 V/m	>0.1 <0.18 V/m	>0.06 <0.16 V/m	>0.08 <0.17 V/m
20 (lPPC/rSOR-35 cm^2^-2 mA)	>0.16 <0.34 V/m	>0.14 <0.25 V/m	>0.17 <0.26 V/m	>0.19 <0.29 V/m	>0.16 <0.27 V/m	>0.18 <0.29 V/m
**Electrode Configuration**	**OFC (S)**	**OFC (C)**	**APC (S)**	**APC (C)**	**STL (S)**	**STL (C)**
1 (lM1/rSOR-20 cm^2^-1 mA)	>0.05 <0.13 V/m	>0.07 <0.16 V/m	>0.08 <0.129 V/m	>0.08 <0.13 V/m	>0.08 <0.16 V/m	>0.1 <0.17 V/m
2 (lM1/rSOR-20 cm^2^-2 mA)	>0.12 <0.27 V/m	>0.17 <0.3 V/m	>0.13 <0.26 V/m	>0.18 <0.27 V/m	>0.18 <0.29 V/m	>0.18 <0.31 V/m
3 (lM1/rM1-20 cm^2^-1 mA)	>0.05 <0.1 V/m	>0.04 <0.08 V/m	>0.12 <0.15 V/m	>0.1 <0.17 V/m	>0.08 <0.17 V/m	>0.08 <0.12 V/m
4 (lM1/rM1-20 cm^2^-2 mA)	>0.1 <0.2 V/m	>0.1 <0.15 V/m	>0.24 <0.36 V/m	>0.2 <0.35 V/m	>0.18 <0.29 V/m	>0.15 <0.25 V/m
5 (lM1/rSOR-35 cm^2^-1 mA)	>0.07 <0.14 V/m	>0.08 <0.15 V/m	>0.09 <0.14 V/m	>0.07 <0.15 V/m	>0.08 <0.15 V/m	>0.07 <0.17 V/m
6 (lM1/rSOR-35 cm^2^-2 mA)	>0.16 <0.29 V/m	>0.16 <0.29 V/m	>0.16 <0.29 V/m	>0.15 <0.29 V/m	>0.16 <0.29 V/m	>0.16 <0.32 V/m
7 (lM1/rM1-35 cm^2^-1 mA)	>0.06 <0.1 V/m	>0.06 <0.09 V/m	>0.08 <0.15 V/m	>0.09 <0.17 V/m	>0.07 <0.13 V/m	>0.06 <0.13 V/m
8 (lM1/rM1-35 cm^2^-2 mA)	>0.12 <0.19 V/m	>0.12 <0.15 V/m	>0.15 <0.31 V/m	>0.17 <0.32 V/m	>0.13 <0.27 V/m	>0.12 <0.25 V/m
9 (lDLPFC/rSOR-20 cm^2^-1 mA)	>0.11 <0.2 V/m	>0.09 <0.26 V/m	>0.03 <0.09 V/m	>0.06 <0.12 V/m	>0.03 <0.08 V/m	>0.04 <0.13 V/m
10 (lDLPFC/rSOR-20 cm^2^-2 mA)	>0.19 <0.39 V/m	>0.18 <0.5 V/m	>0.07 <0.17 V/m	>0.1 <0.21 V/m	>0.08 <0.18 V/m	>0.09 <0.25 V/m
11 (lDLPFC/rDLPF-20 cm^2^-1 mA)	>0.09 <0.2 V/m	>0.07 <0.21 V/m	>0.03 <0.08 V/m	>0.04 <0.11 V/m	>0.05 <0.08 V/m	>0.05 <0.11 V/m
12 (lDLPFC/rDLPF-20 cm^2^-2 mA)	>0.18 <0.39 V/m	>0.13 <0.4 V/m	>0.06 <0.16 V/m	>0.07 <0.19 V/m	>0.09 <0.16 V/m	>0.1 <0.2 V/m
13 (lDLPFC/rSOR-35 cm^2^-1 mA)	>0.12 <0.18 V/m	>0.1 <0.19 V/m	>0.02 <0.08 V/m	>0.05 <0.11 V/m	>0.04 <0.08 V/m	>0.05 <0.1 V/m
14 (lDLPFC/rSOR-35 cm^2^-2 mA)	>0.21 <0.36 V/m	>0.21 <0.39 V/m	>0.09 <0.15 V/m	>0.1 <0.2 V/m	>0.08 <0.16 V/m	>0.09 <0.19 V/m
15 (lDLPFC/rDLPF-35 cm^2^-1 mA)	>0.12 <0.18 V/m	>0.09 <0.17 V/m	>0.05 <0.1 V/m	>0.04 <0.09 V/m	>0.04 <0.07 V/m	>0.04 <0.08 V/m
16 (lDLPFC/rDLPF-35 cm^2^-2 mA)	>0.24 <0.38 V/m	>0.18 <0.34 V/m	>0.1 <0.18 V/m	>0.09 <0.18 V/m	>0.08 <0.15 V/m	>0.09 <0.16 V/m
17 (lPPC/rSOR-20 cm^2^-1 mA)	>0.05 <0.11 V/m	>0.08 <0.18 V/m	>0.1 <0.19 V/m	>0.09 <0.21 V/m	>0.1 <0.19 V/m	>0.08 <0.17 V/m
18 (lPPC/rSOR-20 cm^2^-2 mA)	>0.09 <0.23 V/m	>0.12 <0.31 V/m	>0.19 <0.34 V/m	>0.18 <0.41 V/m	>0.19 <0.35 V/m	>0.13 <0.32 V/m
19 (lPPC/rSOR-35 cm^2^-1 mA)	>0.05 <0.12 V/m	>0.07 <0.13 V/m	>0.09 <0.19 V/m	>0.08 <0.17 V/m	>0.1 <0.18 V/m	>0.08 <0.13 V/m
20 (lPPC/rSOR-35 cm^2^-2 mA)	>0.11 <0.23 V/m	>0.13 <0.29 V/m	>0.18 <0.34 V/m	>0.14 <0.34 V/m	>0.19 <0.35 V/m	>0.13 <0.27 V/m
**Electrode Configuration**	**M1 (S)**	**M1 (C)**	**DLPFC (S)**	**DLPFC (C)**	**PPC (S)**	**PPC (C)**
1 (lM1/rSOR-20 cm^2^-1 mA)	ROI	ROI	>0.13 <0.21 V/m	>0.11 <0.19 V/m	>0.07 <0.16 V/m	>0.07 <0.17 V/m
2 (lM1/rSOR-20 cm^2^-2 mA)	ROI	ROI	>0.26 <0.39 V/m	>0.21 <0.38 V/m	>0.17 <0.32 V/m	>0.15 <0.36 V/m
3 (lM1/rM1-20 cm^2^-1 mA)	ROI	ROI	>0.08 <0.2 V/m	>0.08 <0.17 V/m	>0.08 <0.17 V/m	>0.07 <0.14 V/m
4 (lM1/rM1-20 cm^2^-2 mA)	ROI	ROI	>0.17 <0.38 V/m	>0.14 <0.31 V/m	>0.14 <0.31 V/m	>0.14 <0.29 V/m
5 (lM1/rSOR-35 cm^2^-1 mA)	ROI	ROI	>0.1 <0.17 V/m	>0.09 <0.16 V/m	>0.08 <0.15 V/m	>0.07 <0.13 V/m
6 (lM1/rSOR-35 cm^2^-2 mA)	ROI	ROI	>0.15 <0.37 V/m	>0.19 <0.33 V/m	>0.16 <0.29 V/m	>0.14 <0.28 V/m
7 (lM1/rM1-35 cm^2^-1 mA)	ROI	ROI	>0.07 <0.17 V/m	>0.07<0.17 V/m	>0.07<0.13 V/m	>0.07 <0.14 V/m
8 (lM1/rM1-35 cm^2^-2 mA)	ROI	ROI	>0.16 <0.36 V/m	>0.14 <0.31 V/m	>0.13 <0.29 V/m	>0.14 <0.29 V/m
9 (lDLPFC/rSOR-20 cm^2^-1 mA)	>0.05 <0.15 V/m	>0.05 <0.14 V/m	ROI	ROI	>0.02 <0.06 V/m	>0.05 <0.07 V/m
10 (lDLPFC/rSOR-20 cm^2^-2 mA)	>0.1 <0.27 V/m	>0.1 <0.27 V/m	ROI	ROI	>0.04 <0.11 V/m	>0.09 <0.16 V/m
11 (lDLPFC/rDLPF-20 cm^2^-1 mA)	>0.06 <0.15 V/m	>0.06 <0.14 V/m	ROI	ROI	>0.02 <0.05 V/m	>0.04 <0.07 V/m
12 (lDLPFC/rDLPF-20 cm^2^-2 mA)	>0.11 <0.27 V/m	>0.11 <0.29 V/m	ROI	ROI	>0.04 <0.13 V/m	>0.06 <0.13 V/m
13 (lDLPFC/rSOR-35 cm^2^-1 mA)	>0.05 <0.14 V/m	>0.05 <0.12 V/m	ROI	ROI	>0.02 <0.05 V/m	>0.03 <0.07 V/m
14 (lDLPFC/rSOR-35 cm^2^-2 mA)	>0.09 <0.25 V/m	>0.09 <0.23 V/m	ROI	ROI	>0.05 <0.1 V/m	>0.05 <0.12 V/m
15 (lDLPFC/rDLPF-35 cm^2^-1 mA)	>0.05 <0.14 V/m	>0.05 <0.13 V/m	ROI	ROI	>0.03 <0.06 V/m	>0.03 <0.07 V/m
16 (lDLPFC/rDLPF-35 cm^2^-2 mA)	>0.1 <0.26 V/m	>0.07 <0.25 V/m	ROI	ROI	>0.05 <0.12 V/m	>0.05 <0.15 V/m
17 (lPPC/rSOR-20 cm^2^-1 mA)	>0.08 <0.18 V/m	>0.08 <0.17 V/m	>0.08 <0.19 V/m	>0.08 <0.18 V/m	ROI	ROI
18 (lPPC/rSOR-20 cm^2^-2 mA)	>0.18 <0.35 V/m	>0.13 <0.32 V/m	>0.18 <0.35 V/m	>0.18 <0.36 V/m	ROI	ROI
19 (lPPC/rSOR-35 cm^2^-1 mA)	>0.08 <0.19 V/m	>0.07 <0.17 V/m	>0.08 <0.19 V/m	>0.07 <0.17 V/m	ROI	ROI
20 (lPPC/rSOR-35 cm^2^-2 mA)	>0.18 <0.35 V/m	>0.14 <0.32 V/m	>0.17 <0.35 V/m	>0.14 <0.32 V/m	ROI	ROI

These values were estimated from the color bar showing electric field intensities in the output image of each simulation. APC, anterior-superior parietal cortex; C, COMETS; dmPFC, dorsomedial prefrontal cortex; l/rDLPFC, left/right dorsolateral prefrontal cortex (F3/F4, according to the international 10–20 EEG electrode positioning system); l/rM1, left/right primary motor cortex (C3/C4); lPPC, left posterior parietal cortex (P3); OFC, orbitofrontal cortex; PMC, premotor cortex; ROI, region of interest (for ROI intensity see Table 1); rSOR, right supraorbital ridge (Fp2); S, SimNIBS; STL, superior temporal lobe; vmPFC, ventromedial prefrontal cortex.

## Data Availability

Not applicable.
